# Intake, Nutrient Apparent Digestibility, and Ruminal Constituents of Crossbred Dorper × Santa Inês Sheep Fed Diets with Babassu Mesocarp Flour

**DOI:** 10.1155/2016/8675836

**Published:** 2016-11-13

**Authors:** Osman José de Aguiar Gerude Neto, Michelle de Oliveira Maia Parente, Henrique Nunes Parente, Arnaud Azevedo Alves, Paull Andrews Carvalho dos Santos, Miguel Arcanjo Moreira Filho, Anderson de Moura Zanine, Daniele de Jesus Ferreira, Leilson Rocha Bezerra, Ruan Mourão da Silva Gomes

**Affiliations:** ^1^Department of Animal Science, Federal University of Maranhão, 65500-000 Chapadinha, MA, Brazil; ^2^Department of Animal Science, Federal University of Piauí, 640495-500 Teresina, PI, Brazil

## Abstract

The aim of this study was to evaluate the effect on intake, apparent digestibility, and ruminal constituents of sheep in response to the addition of increasing levels of babassu mesocarp flour (BMF) to the diet. Twenty crossbred sheep (29.17 ± 2.23 kg) were used in a randomized complete block design. Lambs were confined for 21 days, with 16 days for diet adaptation and 5 days for data collection, in which they were fed an isonitrogenous diet (16.5 ± 0.2 CP, DM basis) containing 70% of concentrate and 30% (DM basis) of Tifton 85 hay. Increasing levels of BMF were 0, 10, 20, and 30% (DM basis). There was a quadratic effect (*P* < 0.05) on the DM intake, nutrients intake, and digestibility of CP and NFC. The digestibility of DM, OM, TC, and NDF decreased linearly, while EE digestibility increased linearly with increasing levels of BMF. The high NDF content presented in the chemical composition of the babassu mesocarp flour ranked the same as fibrous food, which can limit the inclusion in the diet of high production animals. So, babassu mesocarp flour is an alternative for energy source in lambs feed and can be added at levels up to 10%.

## 1. Introduction

The formulation of lambs finished in feedlot system is based primarily on corn and soybeans; however, the availability of these grains is variable throughout the year. Uncertain availability thus leads to variations in cost of these raw materials, which directly affects the profitability of lamb production. Therefore, the use of alternative foodstuffs is another way to increase efficiency in animal production, replacing corn and soybeans in feed formulation. In Northeastern Brazil, an important alternative food source is the babassu palm (*Orbignya* ssp.).

Babassu (*Orbignya phalerata* Mart.) is a palm tree native to Brazil's northern states (Piauí, Tocantins, and Maranhão) that appears between the Cerrado and the Amazon rain forest in an ecosystem called Mata dos Cocais [1]. An important feature of babassu byproducts is their availability during the off-seasons of conventional grains, thus making it an important alternative for regional producers [2]. Usually, each tree produces 15–25 bunches of fruit, and each fruit weighs 98–280 g. The average weights of each component of the babassu coconut are 11% exocarp, 23% mesocarp, 59% endocarp, and 7% kernels [3].

The importance of babassu palm tree is related to the production of flour for human consumption, oil source, food, and shade for animals. Furthermore, from mesocarp of babassu coconut is produced the babassu mesocarp flour (BMF), which is offered in the market at low cost. This subproduct has a higher starch content [4]; however there may be contamination when extracting the mesocarp by epicarp and endocarp, which are fibrous, resulting in a BMF with high fiber content and smaller content starch.

A few studies with these byproducts have been conducted, as well as basically existing research that evaluated their nutritional characteristics. In research with cattle, there was no difference in dry matter intake, when increasing levels of babassu mesocarp flour were added on concentrate [5]. Thus, the aim of the present study is to evaluate the effect of increasing levels of babassu mesocarp flour on intake, apparent digestibility, and ruminal constituents of sheep.

## 2. Materials and Methods

### 2.1. The Study Site and Sampling Procedure

The experiment in this study was conducted at the Small Ruminant Sector, Center of Agricultural and Environmental Science, Federal University of Maranhão, Brazil (03°44′33′′S, 43°21′21′′W). Twenty crossbred Dorper × Santa Inês sheep (29.17 ± 2.23 kg initial BW) were confined over a period of 21 days, with 16 days for diet adaptation and 5 days for data collection.

The treatments were defined by the increase in levels of babassu mesocarp flour (Florestas Brasileiras S.A., Itapecuru Mirim, Maranhão, Brazil, Table 1) in the diet. The treatments were as follows: (1) control diet without babassu mesocarp flour (0 BMS); (2) 10% (DM basis) babassu mesocarp flour in the diet (10 BMS); (3) 20% (DM basis) babassu mesocarp flour in the diet (20 BMS); and (4) 30% (DM basis) babassu mesocarp flour in the diet (30 BMS). The experimental diets were formulated according to [6].

### 2.2. Chemical Composition

The composition of the ingredients is shown in Table 1 and the composition of the diets is shown in Table 2.

Corn was coarsely ground using a grinder (Nogueira® DPM-4, Itapira, Brazil) and mixed with soybean meal, babassu mesocarp flour, wheat bran, urea, limestone, and mineral premix. Hay was coarsely chopped to reduce the animal diet selection and feed wastage. The concentrate and Tifton 85 hay were separately weighed using an electronic scale (Marte®, LC 100, São Paulo, SP, Brazil); they were then mixed and offered daily as complete diets. All animals had ad libitum access to feed and fresh water.

On each day of collection, the feed was weighed on a 5 g precision electronic scale and offered ad libitum at 7:30 in the morning. The amount of feed offered was adjusted according to feed intake during the animals' adaptation period, and no orts greater than 10% were allowed. During four different days (17th–20th days) in the collection period, at 7:30 in the morning, the orts were weighed to obtain the dry matter intake (DMI) per animal and the total amount of feces generated in 24 hours. A harness equipped with a bag was used for fecal collection to prevent urine from mixing with feces.

Samples of feed, orts, and feces (10% of the total) were collected during these four days, and composite samples were formed per animal and preserved at −20°C for later analyses. Samples were thawed and dried in a forced-ventilation oven (55°C) for 72 hours and were ground with a Wiley-type mill to be able to pass through a 1 mm screen.

Determination of dry matter (DM), ash, and protein content total was conducted according to [7]. Crude protein (CP) was obtained by multiplying the total N content by 6.25. Organic matter was determined by the difference between DM and ash contents. Neutral detergent fiber and neutral detergent acid corrected for ash (aNDFom and ADF, resp.) were determined according to [8], using heat-stable alpha-amylase and sodium sulfite. The levels of neutral and acid detergent insoluble nitrogen (NDIN and ADIN) were estimated in the waste obtainedafter extraction in neutral detergent and acid, respectively, of the samples through the Kjeldahl procedure [7]. Starch concentrations were measured using the glucogenic assay described by [9, 10].

Nonfibrous carbohydrates (NFC) were estimated according to the equation NFC = 100 − (NDF + CP + EE + ash). Total digestible nutrients (TDN) were calculated according to [11]: TDN = CPdigested + (EEdigested × 2.25) + NDFdigested + NFCdigested. Metabolizable energy (ME) values for each diet were based on the assumption that 1 kg of TDN is equal to 4.409 Mcal of digestible energy (DE) and that 1 Mcal of DE is equal to 0.82 Mcal of ME [6]. The data used to calculate dietary ME were obtained from [12].

The total tannin and condensed tannin contents were determined by the Folin-Ciocalteu method, and the condensed tannin was determined by the method in [13].

### 2.3. Sample of Ruminal Content

Samples of ruminal content were obtained on the 21st day of the experiment, with the first measurement taken prior to feeding. Zero hour was considered the moment before feeding, and measurements were taken then and at 2.5, 5.0, and 7.5 hours after feeding. Rumen ammonia nitrogen (NH_3_-N) and short-chain fatty acids (SCFA) levels in the ruminal fluid were determined.

A representative sample of ruminal content from each animal was collected via esophageal tube [14] where a soft rubber tube with an inside diameter of 1 cm was inserted through the esophagus into the rumen. A vacuum of 1.05 kg/cm* z* was applied to extract the rumen fluid. The first portion of rumen fluid was discarded, and the second portion, after being squeezed through 2 layers of cheesecloth, was used for determining pH, ammonia nitrogen, and short-chain fatty acids (SCFA).

Rumen fluid pH was measured immediately after sampling using a digital pH meter (DM20, Digimed, São Paulo, Brazil). After recording the pH, two 2.5 mL aliquots of rumen fluid were stored in plastic flasks containing 1.25 mL of 6 N HCl and were frozen at −20°C for further analyses of SCFA and ruminal ammonia (NH_3_-N).

To determine SCFA, 1.6 mL of thawed rumen fluid was centrifuged at 15,000 ×g for 15 minutes at 4°C, with 0.4 mL of a 3 : 1 solution of metaphosphoric acid 25% and formic acid 98–100% [15] and 0.2 mL of 2-methyl-butyric 100 mM acid solution (internal standard). After centrifugation, approximately 1.2 mL was transferred to a chromatograph vial for the SCFA separation by GLC (7890-A, Agilent Technologies) equipped with a capillary fused silica column DB-WAX (30 m × 0.25 mm × 0.25 *μ*m of propylene glycol, Agilent Technologies). The total run time was 16.5 min. The external calibration curve was made with chromatograph standards of acetic, propionic, isobutyric, butyric, isovaleric, and valeric acids. The standard mixture of higher concentration (denominated as super high) contained 200 mM of acetic acid, 54 mM of propionic acid, 6 mM of isobutyric acid, 45 mM of butyric acid, 9 mM of isovaleric acid, and 9 mM of valeric acid. The standard solutions were obtained by diluting the super high mixture by 1/2 (high), 1/4 (medium), 1/8 (low), and 1/16 (very low).

The second plastic flask of each sample was thawed for the analysis of NH_3_-N, which was carried out by the colorimetric method described in [16] and adapted to a microplate reader (BIO-RAD), using a filter for absorbance at 550 nm [17].

### 2.4. Experimental Design and Statistical Analysis

The experimental design consisted of a randomized complete block, with four treatments and five blocks per treatment. The blocks were defined according to the weight and age of the animals at the beginning of the experiment. The Shapiro-Wilk normality test was used to check the homogeneity of variances.

Dry matter and nutrient intake, as well as dry matter and nutrient digestibility, were analyzed using the PROC MIXED procedure of [18], according to the model *Y* = *μ* + *B*_*i*_ + *D*_*j*_ + *E*_*ij*_, where *μ* is the overall mean; *B*_*i*_ is the random effect of block; *D*_*j*_ is the fixed effect of diet; and *E*_*ij*_ is the residual error.

Ruminal parameters were analyzed as repeated measures using the PROC MIXED procedure of [15], according to the following statistical model: *Y* = *μ* + *B*_*i*_ + *D*_*j*_ + *S*_*ij*_ + *T*_*k*_ + (*DT*)_*jk*_ + *E*_*ijk*_, where *μ* is the overall mean; *B*_*i*_ is the random effect of block (*i* = 1–5); *D*_*j*_ is the fixed effect of diet; *S*_*ij*_ is the residual error associated with the animal effect (block × diet); *T*_*k*_ is the fixed effect of time; (*DT*)_*jk*_ is the interaction of the diet × time; and *E*_*ijk*_ is the residual error. The covariance matrix that best fit the data set was Autoregressive (AR 1) for pH, NH_3_-N, and C_2_ : C_3_ ratio; Compost Symmetry (CS) for ruminal concentration of acetate, propionate, and isovalerate and total concentration of SCFA; and Nonstructured (NS) for ruminal concentrations of isobutyrate, butyrate, and valerate.

The means were obtained using the LSMEANS command. Orthogonal polynomials for treatment responses were determined by linear and quadratic responses to increasing levels of babassu mesocarp flour incorporation. Effects were declared significant at *P* < 0.05.

## 3. Results and Discussion

### 3.1. Nutrients Intake and Nutrient Digestibility

A quadratic effect (*P* < 0.05) was found on the DM intake and nutrients intake (Table 3). The DM intake of animals fed 30 BMF was lower than that recommended by [6] for sheep of 30 kg of BW which is preconized 1050.0 g·d^−1^.

The BMF is characterized by its fibrous composition (52.7% NDF; 15.8% lignin). It is known that some factors are involved in the regulation of intake in ruminants; among them is the diet NDF content that is considered limiting due to their slow degradation and low pass rate in the rumen. Reference [19] suggested that NDF content is the best single chemical predictor of DM intake by ruminants. The NDF is highly correlated with the volume density of the food, representing the fraction of slow digestion, and therefore is highly correlated with ruminal filling and dry matter intake [20].

A decrease in DM intake also was reported by [4] when addition of BMF was greater than 7.5% in diets of lambs. Reference [21] found a decreasing linear effect on DM intake, when assessing the inclusion (0, 10, 20, and 30%) of babassu meal in the diet of sheep. This result demonstrates that though distinct babassu byproduct was used in this research, it has the same fibrous characteristics, which may affect the dry matter intake due to physical factors of rumen fill. Reference [22] worked with intake and digestibility of lambs fed BMF (41.0% NDF; 20.8% lignin) and also observed reductions on DM and CP digestibility. These results corroborate our data and demonstrate how lignin levels can negatively influence digestibility and promote low degradability of coproducts.

The DM digestibility, OM digestibility, total carbohydrate (TC) digestibility, and NDF digestibility (Table 3) decreased linearly (*P* < 0.05) with increasing levels of BMF which is probably attributed to higher levels of NDF (34.4 to 44.0% in 0 and 30 BMF treatments, resp.) and specially ADF (16.2 to 27.8% in 0 and 30 BMF treatments, resp.). The FDA fraction is composed of cellulose and lignin and digestibility of foods is related to that fraction of the fiber, since lignin (indigestible fiber) has greater proportion of the FDA for the byproduct. Reference [23] studied ruminal fermentation kinetics of flour mesocarps I and II of babassu and stated that they present very lignified fiber fraction, which hardens the colonization of NDF fermentation of feed, because the lignin is indigestible in rumen and is toxic for many ruminal microorganisms.

According to [24], high fiber concentrations in the diet promote ideal conditions for accelerated growth microorganisms that are responsible for the hydrolysis and hydrogenation of dietary fat, which may explain the results found for EE digestibility (Table 3) that increased linearly (*P* < 0.05).

There was observed a quadratic response (*P* < 0.05) to digestibility of CP (Table 3), following the same trend of crude protein intake. This result is probably due to the increase in nitrogen insoluble in detergent neutral and detergent acid in the diets with increasing levels of BMF (Table 1). A quadratic response (*P* < 0.05) was also found for digestibility of NFC. This response is associated with a reduction in NFC intake of animals fed high levels of BMF, resulting in longer permanence of this material in the gastrointestinal tract, that favor the digestibility.

### 3.2. Ruminal Parameters

There was no effect (*P* > 0.05) of increasing levels of BMF on pH, NH_3_-N, propionic acid, and butyric acid (Table 4). There was an effect of feeding time (*P* < 0.05) on all variables analyzed, except for acetate and total SCFA concentration.

**Figure 1:**
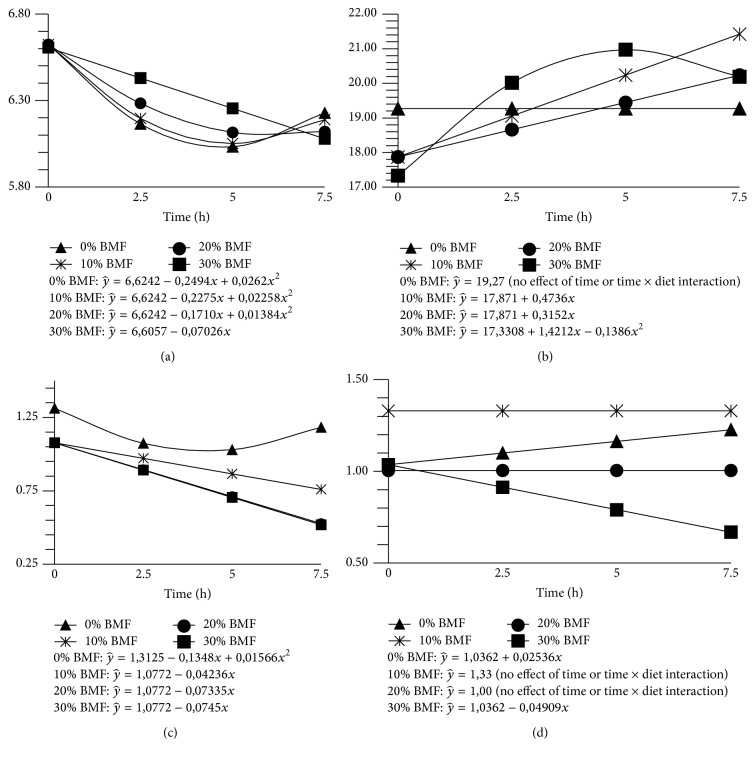
Values of pH (a) and concentration of propionic acid (b), isobutyric acid (c), and valeric acid (d) in ruminal fluid of sheep fed increasing levels of babassu mesocarp flour (BMF).

Although the addition of BMF has reduced DM digestibility, pH remained constant, with values above 6.3. Reference [25] proposed that values of pH below 6.2 would reduce cellulolytic activity. According to [26], maximum activity occurs at pH near 6.5 for most microorganisms. There was interaction (*P* < 0.01) between time and diet for ruminal pH (Table 4, Figure 1). The linear decrease in pH of the rumen fluid over time is related to animals fed diets of higher BMF proportions having the lowest time spent in rumination and a decrease in secretion of saliva [12]; this is different behavior from that observed in the animals fed diets without BMF or with smaller BMF proportion. Furthermore, the higher aNDFom and ADF concentrations in treatment with higher BMF proportions probably reduced the SCFA production speed, contributing to these results.

NH_3_-N values ranged from 19.80 to 22.25 mg·L^−1^. According to [27], maximum fermentative ruminal activity is obtained when NH_3_-N reaches values between 5 and 23 mg/dL. This suggests that, in the current study, there was no ammonia deficiency in the rumen fluid for the synthesis of microbial proteins.

A possible hypothesis for the linear increased effect (*P* < 0.05) on the acetate concentration is related to results obtained from NDF content of diets. This increase in NDF content promotes an increase in acetate concentration (*P* < 0.05) in the rumen of animals that were fed these diets.

There was observed a quadratic effect (*P* < 0.05) for concentrations of propionic acid with increasing levels of BMF, which may be related to lower availability NFC in the rumen of animals fed higher levels of BMF. Also an effect of time and of the interaction of diet × time was observed (*P* < 0.05) (Figure 1). Treatments of 10 BMF and 20 BMF showed a constant growth of propionic acid in the rumen fluid (Figure 1). However, propionic concentration in rumen fluid of animals fed the 30 BMF diet had a quadratic response, decreasing the concentration of these SCFA over time; this may be related to lower NCF intake.

The isobutyric, valeric, and isovaleric acids contents decreased linearly (*P* < 0.05) with increasing BMF levels, probably due to the quadratic response in results obtained with CP intake and CP digestibility. According to [28], branched-chain VFA are derived from the fermentation of branched-chain amino acids. Still, [29] comments that a branched-chain VFA deficiency could be encountered if high-forage diets are low in true protein and NPN is used as a supplement. In this study, the BMF showed low content of crude protein and high NIDA and NIDN which may have contributed to these results.

An interaction effect for diet × time (*P* < 0.05) for isobutyric and valeric acids was observed (Figure 1). Increasing levels of BMF in the diet decreased isobutyric concentration over time, and the lowest mean value was found for the 30 BMF diet, probably due to lower CP digestibility. Isobutyrate and isovalerate are indicative of amino acid fermentation [30], it is produced in the rumen through oxidative deamination of the valine and leucine, respectively [31], and according to [32] these amino acids have slow and medium degradation.

In relation to valeric acid (Figure 1), animals fed with higher levels (20 and 30%) of BMF showed a linear reduction in the presence of this SCFA in rumen fluid; animals fed lower levels (0 and 10%) of BMF showed constant molar concentration of valeric acid. This may be related to the TC digestibility.

The acetate : propionate molar concentration ratio increased with increasing BMF levels, due to the results found for acetate.

## 4. Conclusions

The high NDF content presented in the chemical composition of the babassu mesocarp flour ranked the same as fibrous food, which can limit the inclusion in the diet of high production animals. So, babassu mesocarp flour is an alternative for energy source in lambs feed and can be added at levels up to 10%.

## Figures and Tables

**Table 1:** Chemical composition of ingredients (%, DM basis).

Chemical composition^1^	Tifton hay	Corn	Soybean meal	Wheat bran	Babassu mesocarp flour	Urea	Limestone	Mineral premix
DM	90.1	90.5	90.4	87.8	89.9	100.0	100.0	100.0
OM	95.6	98.6	93.6	94.4	92.7	—	—	—
CP	7.6	8.3	50.5	18.0	4.0	282.0	—	
EE	0.3	2.0	1.7	2.7	0.9	—	—	—
aNDFom	71.4	11.4	20.9	42.9	52.7	—	—	—
NDA	36.7	4.6	10.8	15.0	45.0	—	—	—
NFC	16.4	76.9	20.5	30.7	35.1	—	—	—
TC	87.8	88.3	41.4	73.6	87.8	—	—	—
Lignin					15.8			
NIDN^2^					0.2			
ADIN^2^					0.2			
Starch					30.4			
Total tannin^*∗*^	—	—	—	—	12.5	—	—	—
CT^*∗∗*^	—	—	—	—	6.5	—	—	—

^1^DM: dry matter; OM: organic matter; CP: crude protein; EE: ether extract; aNDFom: neutral detergent fiber corrected for ash; ADF: acid detergent fiber; NFC: nonfiber carbohydrates; TC: total carbohydrates; NDIN: neutral detergent insoluble nitrogen; ADIN: acid detergent insoluble nitrogen; CT: condensed tannin.

^2^% of total N.

^*∗*^Equivalent grams of tannic acid. Kg^−1^ of dry matter.

^*∗∗*^Equivalent grams of leucocyanidin. Kg^−1^ of dry matter.

**Table 2:** Ingredients and chemical composition of experimental diets (%, DM basis).

Ingredients	Babassu mesocarp flour (% of DM)			
	0	10	20	30
Tifton 85 hay	30.0	30.0	30.0	30.0
Corn	43.5	34.9	25.7	18.2
Soybean meal	8.1	9.9	12.7	14.6
Urea	1.3	1.3	1.3	1.3
Limestone	0.8	0.8	0.8	0.8
Mineral premix	1.3	1.3	1.3	1.3
Babassu mesocarp flour	0.0	10.0	20.2	30.2
Wheat bran	14.9	11.8	7.9	3.5

	Chemical composition			
Dry matter	90.3	90.3	91.4	90.5
Organic matter	9.7	9.7	9.7	9.8
Crude protein	16.4	16.3	16.7	16.7
Ether extract	1.5	1.4	1.2	1.1
aNDFom^1^	34.4	37.8	41.0	44.0
NDA	16.2	19.9	23.8	27.8
Nonfiber carbohydrates	44.6	40.9	36.9	33.6
Total carbohydrates	79.1	78.7	77.9	77.6
Total digestible nutrients	79.1	77.0	71.2	69.9
Digestible energy (Mcal·kg^−1^ DM)	3.5	3.4	3.1	3.1
Metabolizable energy (Mcal·kg^−1^ DM)	2.9	2.8	2.5	2.5

^1^aNDF: neutral detergent fiber corrected for ash; ADF: acid detergent fiber corrected for ash.

**Table 3:** Intake (g·day^−1^) and nutrient digestibility (Dig, g·g of DM^−1^) by Dorper × Santa Inês sheep fed increasing levels of babassu mesocarp flour.

Item	Diet^1^				SEM^2^	*P* ^3^		
	0	10	20	30		L	Q	C
Dry matter								
Intake^4^	1157.6	1397.6	1171.2	815.6	0.061	0.005	0.003	0.393
Digestibility^5^	78.8	76.5	70.1	68.8	0.013	0.001	0.826	0.351

Crude protein								
Intake^6^	199.2	239.2	212.4	148.0	0.014	0.016	0.003	0.666
Digestibility^7^	83.8	91.4	83.6	80.0	0.018	0.1707	0.006	0.078

Neutral detergent fiber corrected for ash								
Intake^8^	356.2	490.2	442.8	333.8	0.023	0.484	0.004	0.464
Digestibility^9^	62.7	60.2	47.1	49.9	0.021	0.011	0.518	0.155

Ether extract								
Intake^10^	16.4	17.4	16.0	10.0	0.001	0.001	0.007	0.667
Digestibility^11^	72.4	83.7	84.2	98.2	0.028	<0.001	0.334	0.126

Organic matter								
Intake^12^	1063.2	1273.0	1091.8	770.0	0.063	0.009	0.004	0.495
Digestibility^13^	80.2	77.9	73.3	72.6	0.015	0.004	0.679	0.463

Nonfiber carbohydrates								
Intake^14^	511.6	603.8	470.0	315.2	0.036	<0.001	0.006	0.281
Digestibility^15^	92.6	89.7	95.5	95.7	0.017	<0.001	0.004	0.0601

Total carbohydrates								
Intake^16^	908.0	1094.0	913.0	649.1	0.058	0.006	0.005	0.365
Digestibility^17^	80.9	76.5	72.2	72.3	0.013	0.003	0.254	0.612

^1^Addition of 0, 10, 20, and 30% of babassu mesocarp flour in the diets.

^2^SEM: standard error mean.

^3^ *P*: probability value.

^4^ *Y* = 1174.51 + 3.19*x* − 0.14*x* ^2^; *R* ^2^ = 0.9677.

^5^ *Y* = 79.0834 − 0.3660*x*; *R* ^2^ = 0.9379.

^6^ *Y* = 200.66 + 6.26*x* − 0.261*x* ^2^; *R* ^2^ = 0.9895.

^7^ *Y* = 84.782 + 0.648*x* − 0.0284*x* ^2^; *R* ^2^ = 0.7216.

^8^ *Y* = 362.8 + 17.079*x* − 0.6075*x* ^2^; *R* ^2^ = 0.9584.

^9^ *Y* = 62.7198 − 0.5154*x*; *R* ^2^ = 0.7566.

^10^ *Y* = 16.292 + 0.319*x* − 0.0175*x* ^2^; *R* ^2^ = 0.9882.

^11^ *Y* = 72.9530 + 0.7799*x*; *R* ^2^ = 0.9060.

^12^ *Y* = 1074.7 + 29.262*x* − 1.39*x* ^2^; *R* ^2^ = 0.9753.

^13^ *Y* = 80.1654 − 0.2760*x*; *R* ^2^ = 0.9368.

^14^ *Y* = 521.55 + 11.165*x* − 0.6125*x* ^2^; *R* ^2^ = 0.9689.

^15^ *Y* = 91.8899 − 0.0852*x* + 0.0079*x* ^2^; *R* ^2^ = 0.6797.

^16^ *Y* = 921.5 + 24.17*x* − 1.125*x* ^2^; *R* ^2^ = 0.9633.

^17^ *Y* = 79.9902 − 0.3013*x*; *R* ^2^ = 0.8809.

**Table 4:** Ruminal concentrations of short-chain fatty acids (mol/100 moles), ruminal pH, and ammonia nitrogen (mg·dL^−1^) by crossbred lambs fed diets containing increasing levels of babassu mesocarp flour.

Item^1^	Diets^2^				SEM^3^	*P* ^4^		
	0	10	20	30		L	Q	C
pH^5,6^	6.41	6.39	6.36	6.35	0.035	0.329	0.860	0.091
NH_3_-N (mg/dL)	22.20	22.25	19.83	19.80	0.872	0.033	0.6920	0.2597
Acetate^7^	63.79	63.64	68.05	67.86	0.380	<0.001	0.551	0.078
Propionate^5,8^	20.78	21.55	19.15	19.64	0.262	0.238	<0.001	0.057
Isobutyrate^5,6,9^	1.81	1.36	0.89	0.80	0.071	<0.001	0.738	0.335
Butyrate^5^	9.67	9.90	9.70	8.98	0.181	0.746	0.105	0.584
Isovalerate^5,6,10^	2.92	1.93	1.28	1.02	0.106	<0.001	0.505	0.375
Valerate^5,11^	1.64	1.36	1.00	0.81	0.067	0.001	0.358	0.445
Total (mmol·L^−1^)	72.13	67.18	68.56	71.34	2.11	0.975	0.581	0.874
A : P ratio^12^	3.11	2.96	3.63	3.51	0.058	0.029	0.945	0.053

^1^NH_3_-N: ruminal concentration of ammonia N; A : P: acetate : propionate molar concentration ratio.

^2^Addition of 0, 10, 20, and 30% of babassu mesocarp flour in the diets.

^3^SEM: standard error mean.

^4^Linear, quadratic, and cubic effect.

^5^Hour effect.

^6^Interaction between diet and hour.

^7^ *Y* = 63,525 + 0,1521*x*; *R* ^2^ = 0,7700.

^8^ *Y* = 21.083 − 0.0372*X* − 0.0007*x* ^2^; *R* ^2^ = 0.7082.

^9^ *Y* = 1,7403 − 0,0351*x*; *R* ^2^ = 0,9080.

^10^ *Y* = 2,7431 − 0,0618*x*; *R* ^2^ = 0,8385.

^11^ *Y* = 1,6307 − 0,0291*x*; *R* ^2^ = 0,8975.

^12^ *Y* = 3,0061 + 0,0208*x*; *R* ^2^ = 0,6970.
